# Patterns of longitudinal brain atrophy in the logopenic variant of primary progressive aphasia

**DOI:** 10.1016/j.bandl.2012.12.008

**Published:** 2013-11

**Authors:** Jonathan D. Rohrer, Francesca Caso, Colin Mahoney, Maya Henry, Howard J. Rosen, Gil Rabinovici, Martin N. Rossor, Bruce Miller, Jason D. Warren, Nick C. Fox, Gerard R. Ridgway, Maria Luisa Gorno-Tempini

**Affiliations:** a Dementia Research Centre, Institute of Neurology, University College London, Queen Square, London WC1N 3BG, UK; b Memory and Aging Center, Department of Neurology, UCSF, 350 Parnassus Avenue, Suite 905, San Francisco, CA 94143-1207, United States; c Wellcome Trust Centre for Neuroimaging, UCL Institute of Neurology, Queen Square, London WC1N 3BG, UK

**Keywords:** Primary progressive aphasia, Logopenic aphasia

## Abstract

The logopenic variant of primary progressive aphasia (PPA) is characterised by impaired sentence repetition and word retrieval difficulties. Post mortem studies, amyloid imaging and CSF tau/Aβ measurements suggest Alzheimer’s disease (AD) pathology as the underlying cause. Relatively little is known about patterns of progression in patients with the logopenic variant of PPA. 21 patients (3 with post mortem confirmation of AD and 5 with positive amyloid PIB-PET scans) were studied with longitudinal T1-weighted MR imaging (mean interscan interval 1.2 years) using volumetric analysis and voxel-based morphometry (VBM). Baseline imaging showed asymmetrical (left greater than right) involvement of the posterior superior temporal and inferior parietal lobes as well as posterior cingulate and medial temporal lobes. The whole brain rate of volume loss was 2.0% per year with a greater rate of left hemisphere atrophy (2.3%/year) than right hemisphere (1.6%/year). Longitudinal VBM analysis showed increasing involvement of other areas in the left hemisphere (temporal, parietal, frontal and caudate) and atrophy of areas in the right hemisphere that had been involved earlier in the disease in the left hemisphere, particularly posterior cingulate/precuneus. With disease progression there was worsening of anomia, sentence repetition and sentence comprehension but consistent with the spread of imaging changes also deficits in single word comprehension, single word repetition and verbal memory. This study shows that the logopenic variant of PPA remains an asymmetrical disease, with spread through the left hemisphere language network but also involvement to a lesser degree of regions in the right hemisphere that mirror the earlier left hemisphere changes.

## Highlights

► Patterns of cell loss in lvPPA remain asymmetrical over time. ► More anterior left hemisphere areas become involved over time. ► Right hemisphere regions become affected that mirror early left hemisphere change. ► Left hemisphere atrophy rates are greater than right hemisphere. ► Over time patients with lvPPA develop single word level processing deficits.

## 1 Introduction

The term primary progressive aphasia (PPA) refers to a group of neurodegenerative disorders with language dysfunction as their predominant symptom (Mesulam, 2001). Originally two subtypes, the semantic variant and the nonfluent/agrammatic variant, were described but more recently it has been recognised that there are further subtypes of PPA (Gorno-Tempini et al., 2004, Gorno-Tempini et al., 2008, Rohrer et al., 2010b). Recent consensus guidelines for diagnosis of PPA describe the features of a third subtype, the logopenic variant of PPA or lvPPA (Gorno-Tempini et al., 2011). This disorder has not been as well-studied as the others and unlike the semantic or nonfluent/agrammatic variants which are caused by frontotemporal lobar degeneration pathology (tau or TDP-43), lvPPA appears to be an atypical variant of Alzheimer’s disease (AD), as evidenced by post-mortem studies (Mesulam et al., 2008, Rohrer et al., 2012b), positive amyloid PIB-PET imaging (Leyton et al., 2011, Rabinovici et al., 2008) and a high CSF tau/Aβ ratio (Rohrer et al., 2010a).

A number of cross-sectional imaging studies of lvPPA have now shown a consistent asymmetrical pattern of atrophy with particular emphasis on the areas around the left posterior middle/superior temporal lobe and inferior parietal lobe (temporo-parietal junction) but also the left posterior cingulate, precuneus and medial temporal lobe (Gorno-Tempini et al., 2004, Gorno-Tempini et al., 2008, Migliaccio et al., 2009, Rohrer et al., 2010a, Rohrer et al., 2012b, Wilson et al., 2010). However, few studies have examined longitudinal imaging in lvPPA (Knopman et al., 2009, Rogalski et al., 2011) and little is known about the patterns of change in atrophy over time.

This study aimed to look at longitudinal imaging patterns in lvPPA. We hypothesized (i) rates of whole brain and hemispheric atrophy would be similar to the other PPA subtypes; (ii) over time, there would be increasing involvement of more anterior areas in the left hemisphere; (iii) the contralateral hemisphere would become involved in a pattern similar to that seen first in the left hemisphere.

## 2 Methods

We identified all cases fulfilling consensus criteria for logopenic variant PPA (Gorno-Tempini et al., 2011) with at least two volumetric MRI scans of sufficient quality for analysis from the databases of the Dementia Research Centre at the University College London (UCL) Institute of Neurology and the Memory and Aging Center at the University of California, San Francisco (UCSF). In total, 21 patients were found (9 UCL and 12 UCSF) with three having post-mortem confirmation of Alzheimer’s disease pathology and 5 having positive PIB-PET scans. All patients had undergone detailed clinical and neuropsychological assessment by an experienced behavioural neurologist and any available data on these patients were also extracted from the databases. Ethical approval for the study was obtained from the National Hospital for Neurology and Neurosurgery Local Research Ethics Committee and the UCSF Committee on Human Research.

Subjects were scanned on a 1.5T GE Signa (5), a 1.5T Siemens Magneton Vision (12) or a 3T Siemens Tim Trio (4). Initial image analysis was performed using the MIDAS software (Freeborough, Fox, & Kitney, 1997b). A rapid, semi-automated segmentation technique yielded a brain region separated from surrounding cerebrospinal fluid (CSF), skull and dura. Serial scans were aligned and volume change was calculated directly using the boundary shift integral (BSI) (Freeborough et al., 1997a). BSI-derived whole-brain volume changes (BBSI) were expressed as annualized volume change as a percentage of the baseline brain volume. For all patients and controls we also calculated left and right cerebral hemisphere volumes and rates of atrophy as well as left/right hemisphere volume ratios and rates of change of this hemispheric asymmetry ratio. Scans and associated brain regions were initially transformed into standard space by registration to the Montreal Neurological Institute (MNI) Template. Left and right hemispheric regions were defined using the MNI average brain which was split by dividing the whole volume along a plane coincident with the interhemispheric fissure. An intersection of each individual’s brain region and the hemispheric regions defined on the MNI template was generated to provide measures of brain volume and atrophy in left and right hemispheres. Hemispheric atrophy was expressed as the difference in hemisphere volume between the repeat and baseline scans divided by the baseline hemisphere volume. Annualized rates of hemisphere atrophy were subsequently calculated by dividing by the interscan interval. Data from the lvPPA group were compared against a control group of 20 cognitively normal subjects by looking at the contrasts between the group means using a linear regression model in STATA12 (Stata Corporation, College Station, TX). Wilcoxon signed-rank test was used to look at within-disease group comparisons. There were no significant differences in age, gender, or interscan interval between the groups (Table 1).

**Table 1:** Mean (standard deviation) demographic and baseline volumetric MRI data.

	Controls	lvPPA
Number of subjects	20	21
Male:female ratio	12:8	12:9
Duration of disease (years)	N/A	4.6 (1.6)
Age at baseline scan (years)	63.8 (9.1)	64.4 (7.1)
Interscan interval (years)	1.7 (0.9)	1.2 (0.4)
Baseline brain volume (ml)	1180.6 (96.8)	1061.3 (100.8)⁎
Baseline left hemisphere volume (ml)	581.4 (45.4)	515.1 (49.6)⁎
Baseline right hemisphere volume (ml)	579.5 (47.8)	537.3 (48.7)⁎
Baseline left/right hemisphere ratio	1.00 (0.01)	0.96 (0.02)⁎

⁎ *p* < 0.05 significant difference.

To analyse regional patterns of atrophy cross-sectional voxel-based morphometry (VBM) was performed as previously described (Rohrer et al., 2010a) and detailed in the appendix. A control group of cognitively normal subjects matched for age and gender from both UCL (20) and UCSF (30) were included. We were also interested in assessing longitudinal patterns of change, for which we used a novel method for longitudinal VBM, described in the appendix. Voxel-based data were modelled with factors for group (controls or lvPPA patients, allowing unequal variance), gender (assuming equal variance), and scanner (eight levels, with equal variance assumed), and covariates for age and total intracranial volume (Barnes et al., 2010). For the longitudinal analysis, the logarithm of the interval between the scans was entered as an additional covariate.

## 3 Results

### 3.1 Volumetric imaging

At baseline, brain and hemisphere volumes were significantly smaller in lvPPA than controls. Left hemisphere lobar volumes were significantly smaller than the right with a left/right hemisphere asymmetry ratio significantly lower than the control ratio (Table 1).

**Fig. 1:**
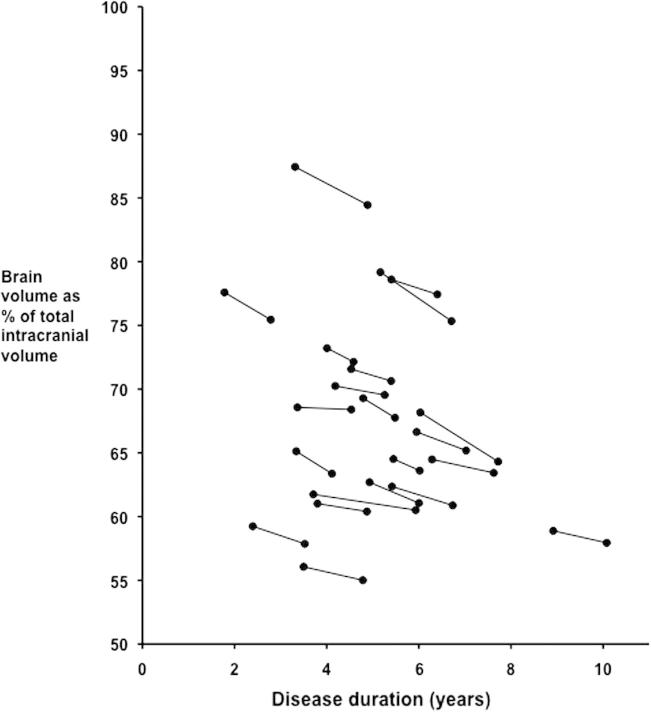
Change in brain volume over time in patients with lvPPA as a function of disease duration.

The rate of whole brain atrophy was significantly greater in lvPPA than controls at 2.0% per year versus 0.3% per year, *p* < 0.05 (Table 2, with individual atrophy rates shown in Fig. 1). Both left and right hemisphere rates of atrophy were greater than controls with the left hemisphere rate (2.3%) greater than the right hemisphere rate (1.6%) (*p* = 0.003). We subsequently went on to use standard methods to calculate sample sizes for detection of a moderate treatment effect (30% reduction in atrophy adjusting for control atrophy rate), including baseline and one follow-up assessment at 12 months with 90% power and 5% two-tailed significance level. For whole brain atrophy rate, the estimated sample size was 69, compared with 107 for the left hemisphere and 165 for the right hemisphere atrophy rate.

**Table 2:** Rates of whole brain and hemispheric atrophy and change in left/right hemisphere ratio.

Outcome measure	Mean rate of atrophy (standard deviation)	
	Controls	lvPPA
Brain BSI (%/year)	0.3 (0.4)	2.0 (0.9)⁎
Left hemisphere (%/year)	0.3 (0.9)	2.3 (1.8)⁎
Right hemisphere (%/year)	0.0 (0.9)	1.6 (1.4)⁎
L/R hemisphere ratio change (%/year)	0.3 (0.7)	0.8 (1.1)

⁎ *p* < 0.05 significant difference, BSI = boundary shift integral.

**Table 3:** Neuropsychometric data from the lvPPA patient group.

	Baseline score Mean (standard deviation)	Annualized decrease in score Mean (standard deviation)
Mini-mental state examination (/30)	20.2 (5.9)	3.9 (3.4)
Boston naming test (%)	61 (26)	18 (10)
Single word repetition (%)	94 (6)	17 (15)
Sentence repetition (%)	46 (32)	26 (23)
Sentence comprehension (US) (%)	66 (19)	9 (10)
Sentence comprehension (UK) (%)	67 (8)	6 (8)
Single word comprehension (US) (%)	94 (7)	9 (6)
Single word comprehension (UK) (%)	77 (19)	5 (15)
Backward digit span	2.8 (1.1)	0.7 (0.7)
Verbal recall memory (US) (%)	38 (19)	7 (9)
Verbal recognition memory (UK) (%)	86 (13)	12 (10)
Calculation (%)	39 (27)	14 (17)

Tests performed for sentence comprehension were CYCLE-R in US (Gorno-Tempini et al., 2004) and PALPA 55 in UK (Kay et al., 1992, Rohrer et al., 2010b), and for single word comprehension were the Pyramids and Palm Trees task in US and the Synonyms task in UK. Verbal recall memory was tested using the California Verbal Learning Test. Verbal recognition memory was tested using the Warrington Recognition Memory Test for Words.

### 3.2 VBM analysis

**Fig. 2:**
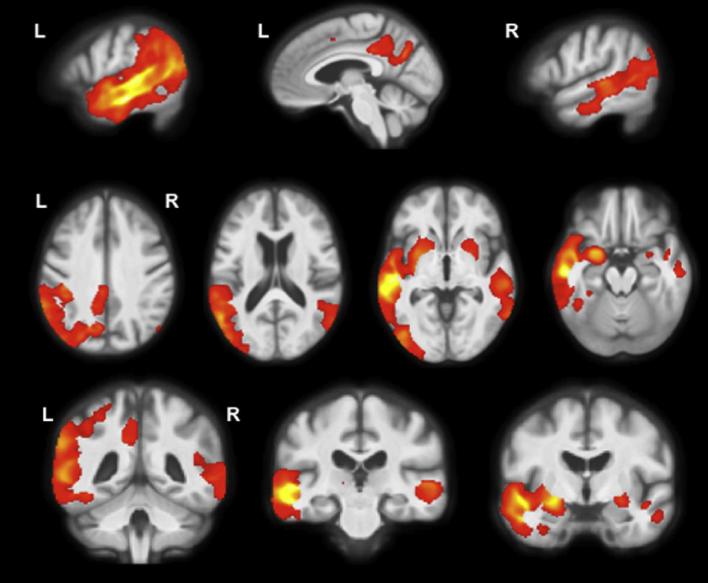
Cross-sectional patterns of grey matter atrophy in lvPPA compared to controls. Statistical parametric maps have been thresholded at *p* < 0.05 after false discovery rate correction over the whole-brain volume and rendered on sagittal (top panel) axial (middle panel) and coronal (bottom panel) sections of a study-specific average group T1-weighted MRI template image in DARTEL space.

Cross-sectional analysis revealed an asymmetrical pattern of grey matter atrophy in the lvPPA group compared with controls with a left-sided predominance (Fig. 2). The most significant areas of grey matter atrophy were in the left posterior temporal lobe (superior and middle temporal gyri), inferior parietal lobe and medial temporal lobe. However, the left posterior cingulate was also involved, as were the right parietal and temporal lobes (mostly posteriorly in the superior and middle temporal gyri and inferior parietal gyrus).

**Fig. 3:**
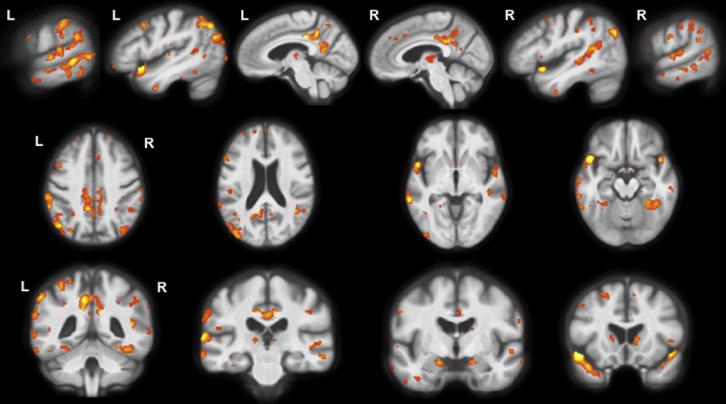
Longitudinal patterns of grey matter atrophy in lvPPA compared to controls. Statistical parametric maps have been thresholded at *p* < 0.05 after false discovery rate correction over the whole-brain volume and rendered on sagittal (top panel) axial (middle panel) and coronal (bottom panel) sections of a study-specific average group T1-weighted MRI template image in DARTEL space.

Longitudinal analysis revealed grey matter atrophy in areas similar to the cross-sectional analysis with asymmetrical left-sided predominant posterior temporal and parietal involvement (Fig. 3, Supplementary Table 1). However there was evidence of posterior cingulate/precuneus involvement both in the left and now the right hemisphere, as well as more anterior and medial temporal lobe involvement, particularly superiorly. Other areas involved to a lesser extent (and not seen in the cross-sectional analysis) included the caudate, the insula and more frontal areas (particularly in the left hemisphere).

### 3.3 Neuropsychometric data (Table 3)

The mean MMSE at baseline was 20.2 (standard deviation 5.9). Consistent with a diagnosis of lvPPA, naming was affected (mean score of 61 (26)% on the Boston Naming test) with sentence repetition more impaired than single word repetition (mean score of 46 (32)% versus 94 (6)%) and sentence comprehension more impaired than single word comprehension. Backwards digit span was decreased with impaired recall but relatively intact recognition memory at baseline as well as evidence of dyscalculia.

Annualized decrease in MMSE was 3.9 (3.4) points and there was a decrease in scores in all tests with worsening of the characteristic deficits of naming, sentence repetition and sentence comprehension as well as recall memory but now also with deficits of single word repetition, single word comprehension and recognition memory.

## 4 Discussion

Here we present global and regional measures of longitudinal neuroimaging in the logopenic variant of PPA, showing that the disorder remains asymmetrical over time with increasing involvement of more anterior areas in the left hemisphere (temporal, frontal and caudate) and a mirroring of the earlier affected left hemisphere regions in the right hemisphere (temporo-parietal junction, posterior cingulate and precuneus).

The rate of brain atrophy was 2.0% per year, similar to that found in a previous study of longitudinal imaging in lvPPA (Knopman et al., 2009). This is a similar rate to that seen in typical early onset AD patients and also in the other PPA variants (Knopman et al., 2009, Rohrer et al., 2012a, Schott et al., 2005). The rate of atrophy of the left hemisphere was greater than that in the right hemisphere with a trend for a greater left/right hemisphere ratio over time compared with controls. This increasing asymmetry is also seen in both the nonfluent/agrammatic and semantic variants of PPA (Brambati et al., 2009, Rohrer et al., 2012a), suggesting that within PPA (at least during this phase of the disease) atrophy spread occurs mainly via an intrahemispheric network of connected brain regions, rather than interhemispherically.

Consistent with the volumetric data the VBM analysis shows an asymmetrical pattern of atrophy initially affecting mostly posterior cortical regions, areas commonly affected in typical Alzheimer’s disease but in this case predominantly in the left hemisphere rather than symmetrically and bilaterally. The presence of initial atrophy around the left temporo-parietal junction is consistent with evidence from stroke aphasias and modern neurocomputational models of language (e.g. Ueno, Saito, Rogers, & Lambon Ralph, 2011) which suggest that damage to this area leads to the development of a conduction aphasia, a presentation similar to many patients with lvPPA. The longitudinal analysis shows that over time there is change in the already affected left posterior superior temporal and inferior parietal regions but also more anterior involvement within the left hemisphere. Interestingly the key areas affected in the right hemisphere are those shown in this and other studies to be the earliest involved left hemisphere areas i.e. temporo-parietal junction, posterior cingulate and medial temporal (Migliaccio et al., 2009, Rogalski et al., 2011).

The key initial neuropsychometric deficits in lvPPA have been described as impaired phonological working memory (poor digit span, sentence repetition and sentence comprehension) and impaired word retrieval (word-finding difficulties in spontaneous speech and anomia). This study supports these results, particularly in finding worse sentence level compared to single word level processing in repetition and comprehension at baseline. One outstanding question is whether this represents a specific impairment in sentence level processing or whether it is due to the intrinsic increased difficulty in performing such tasks compared to single word level processing. It will be useful for future prospective studies of the neuropsychological deficits in lvPPA to compare across PPA phenotypes including those with the semantic and nonfluent variants.

Little is known of the longitudinal cognitive changes that occur in lvPPA with disease progression. In this study there was worsening of the characteristic sentence level processing deficits in association with single word comprehension and repetition deficits and verbal memory impairment. This could be due to increasing atrophy in areas already affected around the left temporo-parietal junction, but is also consistent with the spread of atrophy to more anterior fronto-temporal areas. The involvement of the anterior temporal lobe over time suggests that patients may develop verbal semantic impairment similar to semantic variant PPA – longitudinal studies of single word and semantic processing in lvPPA will be useful to understand this in more detail.

There is a longstanding literature describing patients with Alzheimer’s disease presenting with aphasia (e.g. Clark et al., 2003, Green et al., 1990, Greene et al., 1996, Karbe et al., 1993, Li et al., 2000, Pogacar and Williams, 1984, Snowden et al., 2007, Josephs et al., 2008). It is likely that many of the patients described as having an aphasic presentation of AD have a clinical syndrome equivalent to lvPPA. However, these represent two different levels of description – the presenting clinical syndrome (aphasia) and the likely underlying pathology (Alzheimer’s disease). Whilst evidence suggests that it is likely that most patients with the lvPPA clinical syndrome have underlying Alzheimer’s pathology this is not true for all cases (e.g. Mesulam et al., 2008). Similarly most cases of Alzheimer’s pathology with an aphasic presentation are likely to have an lvPPA syndrome but other clinical presentations have been reported (e.g. Chow et al., 2010, Josephs et al., 2008). Previous studies do show that the imaging features of patients with lvPPA differ from those of typical amnestic AD in that although similar posterior cortical and medial temporal areas are atrophic early on, initially this is very asymmetrical affecting the left more than the right hemisphere (Migliaccio et al., 2009, Ridgway et al., 2012). It remains unclear why this should occur and whether as the disease progresses it starts to look more like typical early onset AD. This study adds to the literature in that it suggests that (a) lvPPA remains asymmetrical at least during the time in which these patients were studied and (b) although other cognitive domains (particularly verbal memory and parietal lobe deficits such as calculation) become involved with disease progression language remains the major clinical feature, placing it firmly within the realm of the primary progressive aphasias.
