# Supplementary material for: Patterns of longitudinal brain atrophy in the logopenic variant of primary progressive aphasia
Source: Brain Lang. 2013 Nov;127(2):121–6. doi: 10.1016/j.bandl.2012.12.008 (PMC3880853; doi:10.1016/j.bandl.2012.12.008)
Supplement: Supplementary data 1 [file mmc1.doc]

**Appendix: Cross-sectional and longitudinal VBM processing**

Methods for applying VBM to serial imaging data are typically modified to take advantage of the fact that smaller changes within-subject can be modelled more precisely than the large inter-subject variability. However, recent papers have highlighted a potential danger of introducing bias to the measurement of temporal change due to asymmetries in longitudinal processing (Thomas et al, 2009; Yushkevich et al, 2010; Fox et al, 2011). Here, we avoid asymmetries in the algorithm by registering (first rigidly aligning, then non-rigidly warping) multiple time-points to their within-subject average. Computation of the initial average requires that the images be approximately aligned; to achieve this without any temporal asymmetry, we reorient all images independently to MNI space.

Initially, all images (both time-points from all subjects) were independently processed with the segmentation algorithm in the “New Segment” toolbox (described in Appendix A of Weiskopf et al, 2011; see also Ashburner et al, 2005). Default settings were used, in SPM8 revision 4290. To standardise intensities, each bias corrected image was divided by its weighted mean intensity, where the weights were the probabilistic WM segmentation. The bias corrected image and the native space tissue segments were then rigidly aligned to MNI space. Next, the probabilistic segmentations for GM, WM and CSF were summed to create an intracranial mask for each image, which were then averaged over the time-points within-subject, resliced to 1mm isotropic, and filtered to remove isolated voxels and expand the mask slightly. The multiple time-points are approximately aligned by virtue of their reorientation to MNI space; however, to register them together precisely, three iterations of realignment to the mean image were performed (using spm_realign, with the probabilistic intracranial segment as a weighting image for the objective function). Each time, the rigid transformations were derived by realigning images that were resliced to 1mm isotropic, but then applied to the original images before reslicing again (so as to avoid the accumulation of interpolation errors). Finally, the images were multiplied with the probabilistic intracranial mask.

Following the initial processing, rigidly aligned and skull-stripped time-points were non-rigidly warped to their average, and this procedure was iterated three more times. SPM’s high-dimensional warping was used, producing one “Jacobian” map per image, giving the relative volume of each voxel in each time-point with respect to the average image for each subject. Logarithms (to base 2) were taken of the Jacobian maps, to make them more normally distributed.

The average images were then segmented using the New Segment toolbox for a second time, producing segmentations in both the resolution of the average (1mm isotropic), and a resolution of 1.5mm isotropic for use in the DARTEL image registration toolbox (Ashburner, 2007). DARTEL was used to warp the GM and WM segments of the within-subject averages to their iteratively evolving between-subject group-wise average (Ashburner et al, 2009). DARTEL transformations were then applied to the 1mm isotropic segmentations (both with and without volume-preserving “modulation”), the average images and log-transformed Jacobian maps (without modulation).

The modulated warped tissue segments were smoothed with a Gaussian kernel of 8mm full width at half maximum (FWHM), these images, derived from the within-subject averages of each subject, were then used for the cross-sectional VBM analysis.

For the longitudinal VBM analysis, the unmodulated warped tissue segments were binarised at a threshold of 0.5 and used for a masked Gaussian smoothing of the warped log-transformed Jacobian maps, similar to the “t-spoon” approach of Lee et al. (2009); i.e. the Jacobian maps were multiplied with the masks and smoothed, then divided by the smoothed masks. The kernel for the finer-scale longitudinal changes was 6mm FWHM. Finally, the differences between these images for the two time-points were divided by the interval in years (equivalent to taking the estimated slopes from within-subject simple regression against time) to provide images for the statistical analysis.

Both cross-sectional and longitudinal statistical analyses were performing using the “flexible factorial” method in SPM8, with analysis restricted to masks defined by binarising the means of the smoothed warped GM segmentations at thresholds determined to maximise correlation between each average and its resultant binary mask (Ridgway et al, 2009). Results are overlaid on a study-specific template defined as the mean of the warped within-subject averages.

**REFERENCES**

1. Ashburner J. [A fast diffeomorphic image registration algorithm.](http://www.ncbi.nlm.nih.gov/pubmed/17761438) Neuroimage. 2007 Oct 15;38(1):95-113.
2. Ashburner J, Friston KJ. [Unified segmentation.](http://www.ncbi.nlm.nih.gov/pubmed/15955494) Neuroimage. 2005 Jul 1;26(3):839-51.
3. Ashburner J, Friston KJ. [Computing average shaped tissue probability templates.](http://www.ncbi.nlm.nih.gov/pubmed/19146961) Neuroimage. 2009 Apr 1;45(2):333-41
4. Fox NC, Ridgway GR, Schott JM. Algorithms, atrophy and Alzheimer's disease: cautionary tales for clinical trials. Neuroimage. 2011 Jul 1;57(1):15-8.
5. Lee JE, Chung MK, Lazar M, DuBray MB, Kim J, Bigler ED, Lainhart JE, Alexander AL. [A study of diffusion tensor imaging by tissue-specific, smoothing-compensated voxel-based analysis.](http://www.ncbi.nlm.nih.gov/pubmed/18976713) Neuroimage. 2009 Feb 1;44(3):870-83.
6. [Ridgway GR](http://www.ncbi.nlm.nih.gov/pubmed?term="Ridgway GR"%5BAuthor%5D), [Omar R](http://www.ncbi.nlm.nih.gov/pubmed?term="Omar R"%5BAuthor%5D), [Ourselin S](http://www.ncbi.nlm.nih.gov/pubmed?term="Ourselin S"%5BAuthor%5D), [Hill DL](http://www.ncbi.nlm.nih.gov/pubmed?term="Hill DL"%5BAuthor%5D), [Warren JD](http://www.ncbi.nlm.nih.gov/pubmed?term="Warren JD"%5BAuthor%5D), [Fox NC](http://www.ncbi.nlm.nih.gov/pubmed?term="Fox NC"%5BAuthor%5D). Issues with threshold masking in voxel-based morphometry of atrophied brains. [Neuroimage.](http://www.ncbi.nlm.nih.gov/pubmed?term=ridgway omar) 2009 Jan 1;44(1):99-111.
7. Thomas AG, Marrett S, Saad ZS, Ruff DA, Martin A, Bandettini PA. [Functional but not structural changes associated with learning: an exploration of longitudinal voxel-based morphometry (VBM).](http://www.ncbi.nlm.nih.gov/pubmed/19520171) Neuroimage. 2009 Oct 15;48(1):117-25.
8. Weiskopf N, Lutti A, Helms G, Novak M, Ashburner J, Hutton C. [Unified segmentation based correction of R1 brain maps for RF transmit field inhomogeneities (UNICORT).](http://www.ncbi.nlm.nih.gov/pubmed/20965260) Neuroimage. 2011 Feb 1;54(3):2116-24.
9. Yushkevich PA, Avants BB, Das SR, Pluta J, Altinay M, Craige C; Alzheimer's Disease Neuroimaging Initiative. Bias in estimation of hippocampal atrophy using deformation-based morphometry arises from asymmetric global normalization: an illustration in ADNI 3 T MRI data. Neuroimage. 2010 Apr 1;50(2):434-45.

Supplementary Table 1 Longitudinal VBM results (MNI co-ordinates)

| **Brain region (Brodmann area)** | **x** | **y** | **z** | **T value** |
| --- | --- | --- | --- | --- |
| Left superior temporal gyrus (22, 38) | -51 | 12 | -8 | 6.8 |
|  | -38 | 20 | -30 | 5.8 |
|  | -57 | 4 | -10 | 5.4 |
| Right superior temporal gyrus (22, 38) | 50 | 13 | -12 | 6.4 |
|  | 57 | -4 | -6 | 4.1 |
| Left cingulate gyrus (30, 31) | -7 | -29 | 37 | 5.9 |
|  | -19 | -58 | 13 | 5.5 |
|  | -8 | -45 | 43 | 5.4 |
| Left middle temporal gyrus (39) | -52 | -73 | 27 | 5.6 |
| Left superior temporal gyrus (42) | -69 | -26 | 8 | 5.5 |
| Left middle temporal gyrus (21) | -66 | -16 | 2 | 5.4 |
| Left inferior parietal lobe (39, 40) | -47 | -63 | 41 | 5.4 |
|  | -58 | -44 | 47 | 5.1 |
| Right inferior parietal lobe (39) | 45 | -73 | 35 | 5.0 |
| Left parahippocampal gyrus | -16 | -4 | -19 | 4.9 |
| Right superior temporal gyrus (13, 39) | 46 | -46 | 13 | 4.7 |
|  | 44 | -54 | 22 | 4.4 |
| Left insula (13) | -44 | -9 | -3 | 4.7 |
| Left inferior temporal gyrus (20) | -59 | -15 | -37 | 4.7 |
| Right parahippocampal gyrus (37) | 31 | -40 | -17 | 4.5 |
|  | 31 | -48 | -13 | 4.4 |
| Left fusiform gyrus (37) | -35 | -45 | 61 | 4.5 |
| Right caudate | 13 | 12 | 6 | 4.5 |
| Right fusiform gyrus (37) | 42 | -45 | -17 | 4.4 |
| Left caudate | -15 | 14 | 7 | 4.3 |
| Right precuneus (19) | 40 | -75 | 45 | 4.3 |
| Right middle temporal gyrus (21) | 60 | -17 | -21 | 4.3 |
| Left precentral gyrus (6) | -55 | 1 | 38 | 4.2 |
